# Sociodemographic and Clinical Factors Impact Non‐Live Vaccine Coverage After Pediatric Solid Organ Transplantation: A Single Center Study

**DOI:** 10.1111/petr.70302

**Published:** 2026-03-23

**Authors:** Adam Z. Blatt, Yunfei Wang, Yeh‐Chung Chang, Sarah M. Heston

**Affiliations:** ^1^ Levine Children's Hospital USA; ^2^ Duke University Human Vaccine Institute USA; ^3^ Department of Pediatrics The University of Texas Southwestern Medical Center USA; ^4^ Department of Pediatrics Duke University USA

**Keywords:** pediatric, solid organ, transplant, vaccines

## Abstract

**Background:**

Pediatric solid organ transplant (SOT) recipients are at increased risk of vaccine preventable diseases (VPD) due to both under‐vaccination and ineffective responses to vaccines while immunosuppressed. Current guidelines recommend timely post‐transplant immunization with non‐live vaccines and surveillance of vaccine‐specific titers to assess vaccine responses; however, institutional adherence to these recommendations may be variable.

**Methods:**

This single‐center retrospective study of 199 pediatric SOT recipients (59 heart, 10 intestinal/multi‐visceral, 34 kidney, and 96 liver) evaluated guideline adherence to post‐vaccine serologic monitoring and identified sociodemographic and clinical factors associated with delayed and incomplete schedules for routine childhood non‐live vaccines after transplant.

**Results:**

Serologic monitoring was utilized after only 8% of recommended vaccines, while participants' age at transplant (odds ratio [OR], 95% confidence interval [CI]: 0.86, 0.81–0.91), receipt of a heart transplant (OR, 95% CI: 0.32, 0.17–0.60), and coverage with private insurance (OR, 95% CI: 0.46, 0.25–0.85) were factors negatively associated with timely initiation or completion of non‐live vaccines after transplant.

**Conclusions:**

The associations between age at transplant, heart transplantation, and type of insurance with under‐immunization warrant further investigation to address modifiable gaps in vaccine coverage and ensure pediatric SOT recipients are optimally protected from VPD.

## Graphical abstract

This single‐center retrospective study of pediatric solid organ transplant recipients found that vaccine‐specific serologies were rarely utilized to guide post‐transplant vaccination practices. Additionally, older age at the time of transplant, private insurance, and heart transplantation were identified as risk factors for delayed and incomplete non‐live vaccine schedules after transplant.

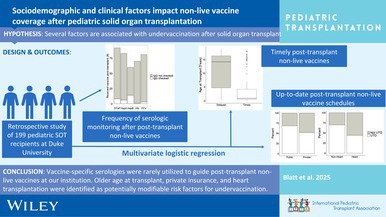

**Abbreviations**
aOR: adjusted odds ratio
AST: American Society of Transplantation
ATG: anti‐thymocyte globulin
CDC: Centers for Disease Control
DEDUCE: Duke Enterprise Data Unified Content Explorer
DTaP: diphtheria tetanus and acellular pertussis
EMR: electronic medical record
Hep A: hepatitis A
Hep B: hepatitis B
Hib:
*Haemophilus influenzae* type B
HPV: human papillomavirus
ICD‐10: International Classification of Diseases Tenth Revision
IPV: inactivated poliovirus
IQR: interquartile range
MCV: meningococcal ACWY conjugate vaccine
NCIR: North Carolina Immunization Registry
OR: odds ratio
PCP: primary care physician
PCV: pneumococcal conjugate vaccine
SOT: solid organ transplant
US: United States
UTD: up‐to‐date
VPD: vaccine preventable disease

## 1 Introduction

Vaccines have been one of the most important public health interventions ever developed, preventing countless illnesses and deaths worldwide and averting trillions of dollars in healthcare costs [1]. Despite the incredible impact of vaccines, pediatric solid organ transplant (SOT) recipients suffer from vaccine preventable diseases (VPD) at increased rates compared to the general population [2, 3, 4, 5]. The reasons for the increased rate of VPD among pediatric SOT recipients are multifactorial. Many pediatric SOT recipients are transplanted at a young age, before they are eligible for many routine childhood vaccinations [6, 7]. After transplant, long‐term immunosuppression impairs the response to vaccines [8, 9, 10, 11, 12, 13]. Transplant complications, psychosocial factors, gaps in communication between medical personnel, and vaccine hesitancy can also lead to delayed post‐transplant vaccine schedules and decreased protection against VPD [14, 15].

Due to the importance of SOT recipient immunization, the Infectious Diseases Community of Practice of the American Society of Transplantation (AST) developed guidance for the vaccination of SOT recipients [16]. While live vaccines are not currently routine among pediatric SOT recipients [17], non‐live vaccines are considered safe for all pediatric SOT recipients and are recommended to begin 3–6 months after transplant [16]. Due to the variability in vaccine responses while receiving immunosuppressive medications, current guidelines suggest monitoring vaccine‐specific serologies to ensure seroprotective responses [16]. The AST guidelines recommend serologic monitoring for tetanus, hepatitis A (Hep A), hepatitis B (Hep B), and *Haemophilus influenzae* type B (Hib), with consideration for pneumococcal conjugate vaccine (PCV) [16, 18]. This strategy guides the need for booster doses after transplant, thus positively impacting vaccine coverage [19].

Despite the initial publication of the AST vaccination guidelines in 2009 [20], it is unknown how well transplant programs adhere to the guideline for serologic monitoring after post‐transplant vaccination. Transplant programs may also have unique policies to guide post‐transplant vaccination, given the current knowledge gaps on the optimal timing of post‐transplant vaccines. Similarly, pediatric transplant programs likely have different resources to ensure early initiation of catch‐up vaccines and completion of vaccine schedules after transplant for their unique populations. Cumulatively, these factors could lead to institution‐ or transplant program‐specific variability in vaccine coverage. Understanding the factors that impede post‐transplant vaccination practices can inform quality improvement efforts and identify areas for further research that could benefit all pediatric SOT recipients. As such, this single‐center retrospective study conducted among pediatric SOT recipients at Duke University between 2014 and 2022 determined adherence to the guidelines for non‐live vaccine serologic monitoring and identified sociodemographic and clinical factors that affected timely initiation and completion of routine non‐live vaccine schedules.

## 2 Methods

### 2.1 Study Population and Design

This was a retrospective, single‐center cohort study of pediatric SOT recipients (< 21 years of age) who underwent transplantation between January 1, 2014, and December 31, 2022. The Duke Enterprise Data Unified Content Explorer (DEDUCE), a data query tool [21], was used to screen the electronic medical record (EMR) for children who were seen by a Duke provider with an International Classification of Diseases, Tenth Revision (ICD‐10) code for solid organ transplantation. The EMR and the North Carolina Immunization Registry (NCIR) were used to verify the receipt of the following non‐live vaccines: diphtheria tetanus and acellular pertussis (DTaP), Hep A, Hep B, Hib, human papillomavirus (HPV), inactivated poliovirus (IPV), meningococcal ACWY conjugate vaccine (MCV), and PCV. Participants were included if they received a transplant at Duke University during the study period or transferred care to Duke within 3 months of transplant received elsewhere within this time frame. Participants were excluded if they died or transferred care to a non‐Duke‐affiliated institution within 6 months post‐transplant, did not have vaccine records that could be verified in the Duke EMR or NCIR, or received chemotherapy within 2 years of transplant. This study was approved by the Duke Institutional Review Board (Pro00112422).

### 2.2 Transplant Practices at Duke University

The immunization history of all children being considered for SOT was reviewed by the transplant team. Catch‐up vaccines were prioritized and administered before the transplant, as time and clinical status allowed. In general, non‐live vaccines were considered at the time of vaccine assessment if the child was not expected to undergo transplantation for at least 2 weeks, and live vaccines were considered if transplantation was not for at least 4 weeks. After transplantation, transplant teams individually assessed patients' readiness to start catch‐up vaccines. Typically, transplant and infectious diseases teams recommended vaccines that were then given by the child's pediatrician. Vaccine serologies were collected at the discretion of the transplant team.

In 2017, the pediatric transplant infectious diseases team was developed and began assisting with pre‐ and post‐transplant vaccine assessments. After 2017, therefore, post‐transplant vaccines were typically recommended by the transplant infectious diseases physician, encouraged by the transplant team, and ultimately administered by the child's local pediatrician.

### 2.3 Study Definitions

For this study, follow‐up time was measured as the time between the day of transplant and the day of the participant's death, transfer of care from Duke, or December 31, 2023—whichever came first. December 31, 2023, was utilized as the end of the study period to ensure that there was at least 1 year of potential follow‐up time for every participant. Participants were considered “eligible” for vaccines if they met at least one of the following criteria: (1) they were not up‐to‐date (UTD) on non‐live vaccines per the Centers for Disease Control (CDC) age‐based schedule prior to transplant [22]; (2) they were UTD on non‐live vaccines at the time of transplant but were due for additional doses per the CDC age‐based schedule in the first 2 years after transplant; or (3) they had a non‐protective vaccine serology obtained pre‐transplant and did not receive any additional doses prior to transplant (Table S1).

When evaluating the initiation of post‐transplant vaccines, this study focused on the first 2 years after the time of transplant. Participants were considered to have received “timely” vaccines if they received a dose of any of the non‐live vaccines (DTaP, Hep A, Hep B, Hib, HPV, IPV, MCV, or PCV) within the first 2 years after transplant, even if the participant did not have a full 2 years of post‐transplant follow‐up time. Participants were considered to have “delayed” vaccines if (1) they had at least 2 years of post‐transplant clinical follow‐up at Duke University and (2) they failed to receive any non‐live vaccines within that time. Participants who had fewer than 2 years of post‐transplant follow‐up time and did not receive any non‐live vaccines during their abbreviated follow‐up time were excluded from the evaluation of timely versus delayed vaccines.

When evaluating the completeness of post‐transplant vaccination, participants were followed for the remainder of the study period, i.e., until the time of the participant's death, transfer of care from Duke, or December 31, 2023—whichever came first. They were considered either “UTD” or “Not UTD” depending on whether they met the age‐appropriate criteria for being UTD (Table S1) for each of the 6 non‐live routine childhood vaccines (DTaP, Hep A, Hep B, Hib, IPV, and PCV) by the end of their follow‐up time. MCV and HPV vaccine schedules were not considered when making the determination of UTD versus Not UTD, because a minority of participants were age‐eligible for routine administration of these vaccines by the end of their follow‐up time.

Because the vaccine composition was not always reported, the eligibility criteria for Hib were derived from the guidelines for the polyribosylribitol phosphate‐outer membrane protein complex formulation, which requires the fewest number of doses. Similarly, any PCV composition (PCV‐7, PCV‐13, or PCV‐20) was accepted as part of the routine vaccine schedule, and adherence to guidelines for booster doses of pneumococcal polysaccharide vaccine 23‐valent or PCV‐20 was not assessed.

Insurance coverage was classified as public if either Medicaid or Medicare was billed at the time of the initial transplant encounter, even if there were additional insurances linked to the encounter. All other insurance providers were classified as private. Participants who were treated for antibody‐ or cellular‐mediated rejection within the first two years after transplant were considered to have experienced early rejection.

### 2.4 Statistical Analysis

Descriptive statistics are reported for post‐vaccination serological monitoring. To determine the impact of clinical factors on vaccination status, the following were included in statistical analyses: age at transplant, race, sex, ethnicity, date of transplant, organ transplanted, distance between home address and the hospital, type of insurance, anti‐thymocyte globulin (ATG) induction, early rejection, and length of follow‐up time. Chi‐squared tests were used to compare the proportion of categorical variables, and analysis of variance (ANOVA) was used to compare the means of continuous variables between each type of SOT.

Two logistic regression models were built to determine the impact of clinical factors on post‐transplant vaccination. Both models used a binary outcome for vaccination. The first model included only participants considered eligible for post‐transplant non‐live vaccines and determined clinical factors that were associated with timely administration of vaccines in the first 2 years after transplantation. The second model included all participants, even those who were not considered eligible for vaccines within the first 2 years after transplant and identified clinical factors that impacted whether they were UTD for all 6 assessed non‐live vaccines by the end of the study period. Univariable logistic regression was used to identify specific risk factors to include in multivariable models. Forward, backward, and stepwise regression were then used to create the final models for hypothesis testing. To optimize statistical power for the available sample size, the multilevel categorical variable organ transplanted was converted to a binary variable (“heart” vs. “non‐heart”) to distinguish between the two groups of transplant surgeons (cardiothoracic and abdominal) who may influence post‐transplant care. Similarly, race and ethnicity were coded as “white” and “non‐white” and “Hispanic” and “non‐Hispanic,” respectively. SAS v9.4 (SAS Institute Inc., Cary, NC) and R version 4.4.1 were used for all analyses.

## 3 Results

### 3.1 Demographics

The DEDUCE screen identified 232 participants who underwent screening for study eligibility. Thirty‐three participants met exclusion criteria due to having less than 6 months of follow‐up time after transplant, transferring to Duke 3 or more months after transplant elsewhere, not being able to verify vaccine records, or receiving chemotherapy within 2 years of transplant. The remaining 199 participants formed the final cohort (Figure S1). Ninety‐six (48%) participants were liver transplant recipients, with heart transplant recipients constituting the next highest percentage (30%). There were expected differences in the age at transplant between the different organ types with liver (0.9, interquartile range [IQR] 0.6–7.1 years) and kidney (12.4, IQR 7.0–15.6 years) transplant recipients having the lowest and highest median age at the time of transplant, respectively. About half of the participants were male (52%), and they were generally white (41%) and non‐Hispanic (80%). Most participants (62%) had public insurance at the time of transplant. The median distance between participants' home address and the hospital was 81.8 miles, and there were not significant differences between each type of SOT (p‐value 0.66) (Table 1). Immunosuppressive therapy was variable among the cohort. Most heart (88%) and intestinal (70%) transplant recipients received ATG for induction (*p* < 0.001), and intestinal transplant recipients (80%) were more often treated for rejection within the first 2 years after transplant compared to the other SOTs (*p* = 0.05) (Table 1).

**TABLE 1:** Demographics and prevalence of clinical factors by transplanted organ.

Factor	Heart *N* = 59	Intestine *N* = 10	Kidney *N* = 34	Liver *N* = 96	Total *N* = 199	*p* ^a^
Female	31 (53%)	6 (60%)	13 (38%)	46 (48%)	96 (48%)	0.50
Race						0.76
American Indian or Alaska Native	4 (7%)	0 (0%)	1 (3%)	3 (3%)	8 (4%)	
Asian	1 (2%)	0 (0%)	3 (9%)	4 (4%)	8 (4%)	
Black or African American	21 (36%)	2 (20%)	8 (24%)	31 (32%)	62 (31%)	
Native Hawaiian or other Pacific Islander	0 (0%)	0 (0%)	0 (0%)	1 (1%)	1 (0%)	
Two or more races	0 (0%)	0 (0%)	1 (3%)	2 (2%)	3 (2%)	
Unknown	6 (10%)	3 (30%)	6 (18%)	20 (21%)	35 (18%)	
White	27 (46%)	5 (50%)	15 (44%)	35 (36%)	82 (41%)	
Ethnicity						0.07
Hispanic	4 (7%)	3 (30%)	6 (18%)	21 (22%)	34 (17%)	
Non‐Hispanic	55 (93%)	7 (70%)	27 (79%)	70 (73%)	159 (80%)	
Unknown	0 (0%)	0 (0%)	1 (3%)	5 (5%)	6 (3%)	
Age at transplant, years Median (25%–75%)	9.7 (0.6–15.3)	3.3 (2.1–5.9)	12.4 (7.0–15.6)	0.9 (0.6–7.1)	3.6 (0.7–14.2)	< 0.001^b^
Private insurance	21 (36%)	0 (0%)	12 (35%)	42 (44%)	75 (38%)	0.05
Distance from hospital, miles Median (25%–75%)	79.8 (34.9–121)	88.8 (79.8–117)	78.8 (29.9–144)	83.7 (45.1–130)	81.8 (36.9–131)	0.66^b^
ATG induction	52 (88%)	7 (70%)	8 (24%)	0 (0%)	67 (59%)	< 0.001
Early rejection	23 (39%)	8 (80%)	11 (32%)	45 (47%)	87 (44%)	0.05
Eligible for post‐transplant vaccines	48 (81%)	6 (60%)	17 (50%)	80 (83%)	151 (76%)	

^a^ Chi‐squared test unless specified otherwise.

^b^ ANOVA.

### 3.2 Prevalence of Vaccine‐Specific Serologic Monitoring

**FIGURE 1:**
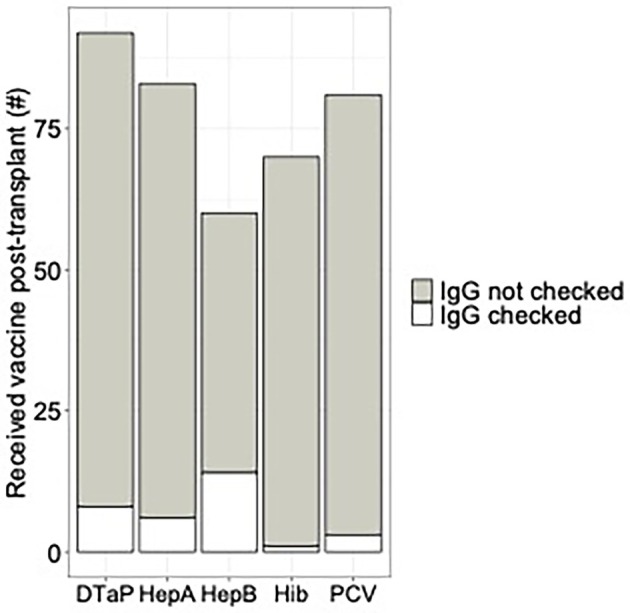
Vaccine‐specific serologies checked after post‐transplant vaccination. The y‐axis represents the number of participants who received at least 1 dose of the designated vaccine after transplant. The number of participants who subsequently had serologies checked for the designated vaccine are indicated in white (IgG checked), while participants who did not have serologies checked are indicated in gray (IgG not checked).

The prevalence of non‐live vaccine serologic monitoring after post‐transplant vaccination was determined to assess adherence to current guidelines. One hundred sixteen participants received at least 1 dose of any of the 5 non‐live vaccines for which serologic monitoring is recommended after transplant: 92 participants received DTaP; 83 received Hep A; 60 received Hep B; 70 received Hib; and 81 received PCV. Since guidelines suggest monitoring serologies for each vaccine independently, there were 386 opportunities for serologic monitoring after post‐transplant vaccination. Vaccine‐specific serologies, however, were assessed in only 25 of 116 participants corresponding to 32 (8%) serologies checked out of the total 386 vaccinations (Figure 1). Hep B titers were checked most frequently (23%), while Hib titers were checked the least (only once among 70 vaccinations). Titers for tetanus (i.e., DTaP) were assessed after 8 (9%) vaccinations, Hep A after 6 (7%), and PCV after 3 (4%). The unexpectedly low adherence to non‐live vaccine‐specific serologic monitoring across all transplant programs indicated that serologies were not used to guide the need for post‐transplant booster doses and precluded further investigation into sociodemographic and clinical factors that affect adherence to this guideline.

### 3.3 Factors Associated With Delayed Initiation of Non‐Live Vaccines After Transplant

Next, multivariable logistic regression was used to identify clinical and sociodemographic factors that impacted timely initiation of non‐live vaccines after transplant. One hundred fifty‐one (76%) participants were eligible to receive non‐live vaccines within the first 2 years after transplant (Table 1). Forty (26%) of 151 eligible participants had delayed non‐live vaccines after transplant. An ATG‐containing induction regimen (odds ratio [OR] = 0.37, 95% confidence interval [CI]: 0.18–0.79), age at transplant (OR = 0.86, 95% CI: 0.81–0.91), and private insurance (OR = 0.45, 95% CI: 0.22–0.94) were negatively associated with timely non‐live vaccines after transplant in univariable analyses; however, only the association with age at transplant remained in the final model after forward, backward, and stepwise regression (Table 2). The median age at transplant of subjects with delayed catch‐up vaccines was 14.2 (IQR: 4.7–16.3) years, while subjects with timely catch‐up vaccines had a median age of 0.9 (IQR: 0.5–4.2) years (Figure S2). These data suggest that older SOT recipients were less likely to receive catch‐up or routine age‐appropriate non‐live vaccines after transplant compared to children transplanted at younger ages when most routine non‐live vaccines are administered.

**TABLE 2:** Factors associated with timely post‐transplant vaccines.

Factor	OR (95% CI)	*p*
**Age at transplant**	**0.86 (0.81–0.91)**	**< 0.001**
Heart transplant	0.52 (0.25–1.11)	0.09
Private insurance	0.45 (0.22–0.94)	0.04
ATG induction	0.37 (0.18–0.79)	0.01
Female	1.33 (0.65–2.75)	0.45
White	0.56 (0.27–1.16)	0.12
Hispanic	1.86 (0.59–5.84)	0.29
Early rejection	0.77 (0.37–1.60)	0.49
Transplant date	1.0001 (0.9996–1.0005)	0.14
Distance	0.9961 (0.9896–1.0027)	0.22

*Note:* The bolded values correspond to the variables that remained in the final model after forward, backward, and stepwise regression.

### 3.4 Factors Associated With Not Being UTD on Non‐Live Vaccine Schedules

Multivariable logistic regression was next used to determine the factors associated with not being UTD on childhood non‐live vaccines, excluding MCV and HPV, by the end of the study period. This analysis included all participants, regardless of whether they were considered eligible for vaccines within the first 2 years after transplant. MCV and HPV were excluded from this analysis since only 93 (47%) participants were at least 11 years of age—the recommended age to start these vaccines—by the end of the study period. In total, 75 of 199 participants (38%) were not UTD on all 6 vaccines. Age at transplant (OR = 0.96, 95% CI: 0.92–1.00), heart transplantation (OR = 0.34, 95% CI: 0.18–0.63), private insurance (OR = 0.50, 95% CI: 0.28–0.90), ATG induction (OR = 0.47, 95% CI: 0.26–0.86), and white race (OR = 0.54, 95% CI: 0.3–0.96) were negatively associated with being UTD, while follow‐up time (OR = 1.0004, 95% CI: 1.0001–1.0007) was positively associated in univariable analyses (Table 3). Heart transplantation (adjusted OR [aOR] = 0.46, 95% CI: 0.25–0.85) and private insurance (aOR = 0.32, 95% CI: 0.17–0.60) were the only factors that remained in the final model, and both were negatively associated with being UTD on all 6 vaccines (Table 3). Twenty‐six (44%) of 59 heart transplant recipients were UTD on all 6 vaccines by the end of the study period, while 98 (70%) of the remaining 140 SOT recipients were UTD. Hep B (71%), Hep A (72%), and DTaP (80%) were the vaccines with the lowest percentage of heart transplant recipients who were UTD (Table S2). Notably, 12 (20%) of 59 heart transplant recipients had negative pre‐transplant Hep B serologies despite having already received at least 3 doses of Hep B and did not receive any additional Hep B doses, making them not UTD (data not shown). Although MCV and HPV were not included in the regression analysis, only 57 (66%) and 50 (60%) of the age‐eligible participants were UTD on MCV and HPV vaccines, respectively (Table S2). Thirty‐nine (52%) of 75 participants with private insurance were UTD on all 6 vaccines, compared with 85 (69%) of 124 participants with public insurance (Figure S3). Taken together, these data indicate that heart transplant recipients and participants with private insurance were more likely to be under‐vaccinated by the end of the study period.

**TABLE 3:** Factors associated with being UTD on all 6 routine childhood non‐live vaccines.

Factors	Univariable analyses		Multivariable final model	
	OR (95% CI)	*p*	aOR (95% CI)	*p*
Age at transplant	0.96 (0.92–1.00)	0.04		
Heart transplant	**0.34 (0.18–0.63)**	**< 0.001**	**0.32 (0.17–0.60)**	**< 0.001**
Private insurance	**0.50 (0.28–0.90)**	**0.02**	**0.46 (0.25–0.85)**	**0.01**
ATG induction	0.47 (0.26–0.86)	0.01		
White	0.54 (0.30–0.96)	0.04		
Female	1.31 (0.74–2.34)	0.35		
Hispanic	1.56 (0.70–3.48)	0.28		
Early rejection	1.17 (0.65–2.09)	0.60		
Transplant date	1.0000 (0.9997–1.0003)	0.80		
Distance	0.9974 (0.9924–1.0024)	0.30		
Follow‐up time	1.0004 (1.0001–1.0007)	0.01		

*Note:* The bolded values correspond to the variables that remained in the final model after forward, backward, and stepwise regression.

## 4 Discussion

This study identified clinical and sociodemographic factors that impacted adherence to post‐transplant vaccination guidelines among pediatric SOT recipients at Duke University. Between 2014–2022 and despite the recommendation to begin live vaccines 6 months after transplant, almost 1 out of every 4 eligible SOT recipients did not receive routine non‐live vaccines even within the first 2 years post‐transplant. Furthermore, nearly 40% of children were not UTD on all 6 routine childhood non‐live vaccines by the end of the study period. Pediatric SOT programs at Duke University did not rely on vaccine‐specific serologic monitoring to guide completion of non‐live vaccine schedules after transplant, as only a handful of vaccinations had the recommended serological monitoring. Children who were older at the time of transplant were more likely to have delays in initiating post‐transplant vaccines, and heart transplant recipients and children with private insurance were less likely to be UTD on all non‐live vaccines. Taken together, these data elucidate factors that may contribute to under‐vaccination in this immunocompromised population and identify key areas that can be addressed to improve the timeliness and completion of vaccine schedules after transplant to optimize vaccine‐mediated immunity.

The age‐related delays in starting non‐live vaccines after transplant are likely multifactorial. Adolescents, in general, are less likely to be UTD on vaccines required at this age, including MCV and HPV [23, 24]. In North Carolina, the percent of children aged 13–17 who had received at least 1 dose of MCV remained below 90% for the first 5 years of the study period (2014–2018), while HPV coverage (at least 1 dose) ranged from as low as ~60% to a peak of ~85% in 2021 [25]. Of the participants who were at least 11 years of age by the end of the study period, over a third were not UTD on either MCV or HPV, suggesting generally poor uptake of these vaccines in the primary care setting could have contributed to the association between age and delayed vaccines.

There is also minimal guidance on the necessity of repeat vaccine doses for non‐protective non‐live vaccine serologies obtained pre‐transplant. As such, it is plausible that non‐protective serologies in older SOT recipients were not considered an indication for vaccination after transplant if vaccine schedules were otherwise complete. For instance, the majority of heart transplant recipients who were not UTD on childhood vaccines had not received any additional doses of Hep B despite negative pre‐transplant serologies. Considering vaccine‐specific antibodies in SOT recipients wane after transplant [26, 27], future studies are needed to evaluate whether vaccine‐specific serologies are reliable predictors of the risk for VPD among pediatric SOT recipients.

Heart transplantation was identified as a risk factor for delayed and incomplete vaccination. Heart transplant recipients typically experience more post‐transplant morbidity and mortality compared to other types of SOT [28, 29]. While an increased frequency of complications could lead to additional medical encounters and therefore vaccination opportunities, data from this study suggest that post‐transplant complications experienced by heart transplant recipients may have disrupted catch‐up vaccine schedules to a greater extent compared with other SOT recipients. Rates of hospitalization for infections, rejection occurring more than 2 years after transplant, or other transplant‐related complications between the different SOT recipients were not assessed in this study; thus, this assertion remains speculative. Qualitative studies to better understand how heart transplant teams perceive the risks of vaccination in the setting of post‐transplant complications could be beneficial in helping to design studies to address barriers to vaccination among heart transplant recipients.

Unexpectedly, SOT recipients with private insurance were less likely to be UTD on routine post‐transplant vaccinations. Over 90% of North Carolina pediatricians are enrolled in the North Carolina Immunization Program that requires reporting to NCIR [30], making differential vaccine reporting to NCIR among PCPs who see publicly versus privately insured children an unlikely explanation. Data from the National Immunization Survey [31, 32] and North Carolina‐specific surveillance [33] demonstrate higher rates of vaccination among children with private insurance compared to those who qualify for Medicaid, thus it is unclear why private insurance negatively affected vaccination rates in this study. Varying levels of confidence in vaccines or different attitudes regarding the necessity of catch‐up vaccines among privately‐ and publicly‐insured families and their primary care physicians (PCPs) is a possible explanation. Ultimately, insurance provider is likely a surrogate marker of more complex risk factors and more studies are needed to corroborate this finding and potentially guide quality improvement efforts to improve vaccine coverage among privately‐insured pediatric SOT recipients.

Importantly, the pediatric transplant infectious diseases service was created during the study period to improve the specialized care of pediatric transplant recipients. We hypothesized that the implementation of this team would impact vaccination practices, and the date of transplant was included in analyses to account for the change in clinical practice. Transplant date, however, was not a significant factor in either model, suggesting that the transplant infectious diseases service did not greatly impact vaccination rates. It is also possible that any positive impact the transplant infectious diseases service imparted on vaccine uptake was countered by other temporal factors, including personnel changes among the transplant teams and the coronavirus disease 2019 pandemic [34]. Regardless, quality improvement efforts to improve vaccine coverage among pediatric SOT recipients at Duke are currently underway.

There are several notable limitations to this work. This study is limited by its retrospective nature and is subject to unmeasured bias. Additionally, serologic responses to non‐live vaccines may be imperfect surrogate measures of protective immunity, and more work is needed to identify accurate markers of vaccine‐mediated immunity among pediatric SOT recipients. However, current expert recommendations encourage measuring serologic response after vaccination, and the goal was to determine guideline‐concordant practices within our institution. Next, at Duke University, pediatric SOT and transplant infectious disease programs provide clearance to begin post‐transplant vaccines, and most vaccines are then administered by the children's PCPs. Gaps in communication between transplant teams and PCPs could not be assessed in this study, nor could the study evaluate provider (transplant team or PCP) attitudes regarding the need to prioritize catch‐up vaccines or to assess serologic response given the overall low prevalence of VPD in the US. Vaccine hesitancy among families was also unable to be assessed in this study, and vaccines administered outside of Duke or at the PCP office, such as at dialysis centers or retail pharmacies, could not be accounted for. Given the traditionally close follow‐up with pediatric transplant providers after transplant, however, outside vaccines were an unlikely source of bias. Further studies are needed to better characterize provider‐ and family‐specific attitudes towards catch‐up vaccines at older ages, following transplant‐related complications, and across different socioeconomic backgrounds.

As general uptake of vaccines continues to decrease across the globe and outbreaks of VPD become more commonplace, it is critical that pediatric SOT recipients, who remain on lifelong immunosuppression, are optimally protected from VPD. This study identified several factors that impacted the timely initiation and completion of vaccine schedules, thus providing areas for targeted interventions to increase vaccine coverage in this vulnerable population. While this was a single center study, the associations between age, heart transplantation, and private insurance with delayed and incomplete vaccine schedules may be generalizable to other institutions. Further prospective multi‐center studies and quality improvement efforts are needed to confirm these findings and improve vaccine uptake among pediatric SOT recipients. Only with a concerted effort can pediatric transplant programs ensure that all pediatric SOT recipients receive optimal vaccine coverage—not just with non‐live vaccines, but also with seasonal and live vaccines—to best protect them from the increasing threat of VPD.

## Funding

This work was supported by the National Institute of Child Health and Human Development (GrantK12HD105253), National Heart, Lung, and Blood Institute (Grant K12HD105253).

## Conflicts of Interest

The authors declare no conflicts of interest.

## Supporting information


**Figure S1:** Flowchart for cohort creation.

**Figure S2:** Age at transplant among participants with delayed vs timely post‐transplant vaccines. Box plots showing the distribution of ages among eligible participants with delayed vs. timely catch‐up vaccines are shown above. Participants were considered to have timely catch‐up vaccines if they started non‐live catch‐up vaccines within 2 years after transplant. Participants were considered to have delayed catch‐up vaccines if they failed to start non‐live catch‐up vaccines within 2 years of transplantation.

**Figure S3:** Proportion of participants UTD for all childhood non‐live vaccines by type of transplant and insurance. The proportion of participants UTD vs. not UTD on all 6 routine non‐live childhood vaccines among heart vs. non‐heart transplant recipients (left panel) and participants with public vs. private insurance (right panel) is shown above. Participants were considered UTD if they met criteria for being UTD for each of the 6 non‐live vaccines. Participants were considered not UTD if they failed to meet criteria for being UTD for at least 1 of the 6 non‐live vaccines.

**Table S1:** Definitions of eligibility and completeness for non‐live vaccines.

**Table S2:** UTD vaccines by transplant type.

## Data Availability

The data that support the findings of this study are available from the corresponding author upon reasonable request.
