# Supplementary figures and images for: Sociodemographic and Clinical Factors Impact Non‐Live Vaccine Coverage After Pediatric Solid Organ Transplantation: A Single Center Study
Source: Pediatr Transplant. 2026 Mar 23;30(3):e70302. doi: 10.1111/petr.70302 (PMC13009305; doi:10.1111/petr.70302)

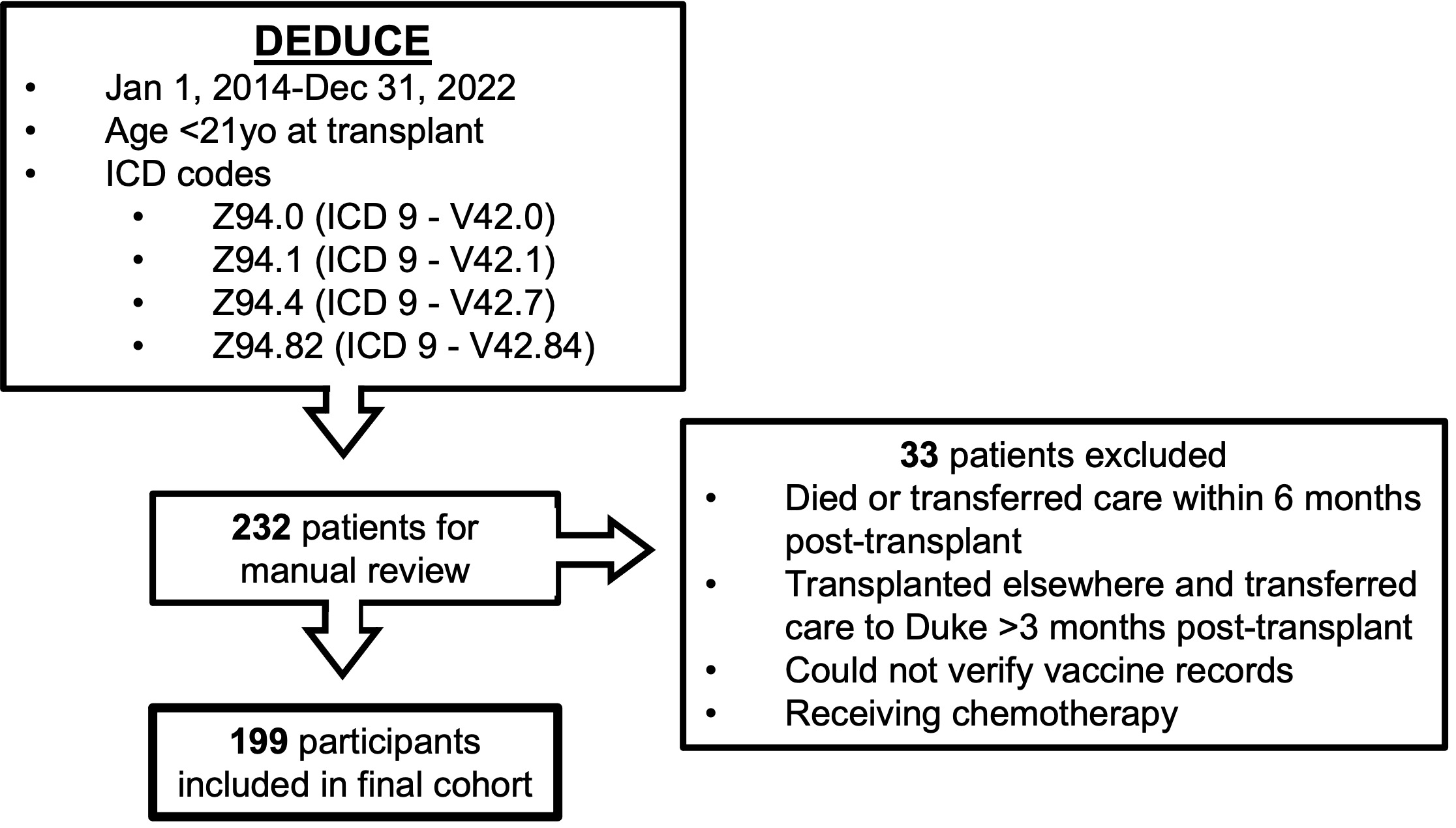

Supplement: Supplementary file 1 — Figure S1: Flowchart for cohort creation. [file PETR-30-e70302-s005.jpg]

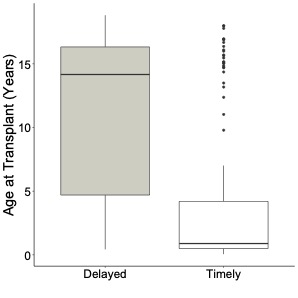

Supplement: Supplementary file 2 — Figure S2: Age at transplant among participants with delayed vs timely post‐transplant vaccines. Box plots showing the distribution of ages among eligible participants with delayed vs. timely catch‐up vaccines are shown above. Participants were considered to have timely catch‐up vaccines if they started non‐live catch‐up vaccines within 2 years after transplant. Participants were considered to have delayed catch‐up vaccines if they failed to start non‐live catch‐up vaccines within 2 years of transplantation. [file PETR-30-e70302-s003.jpg]

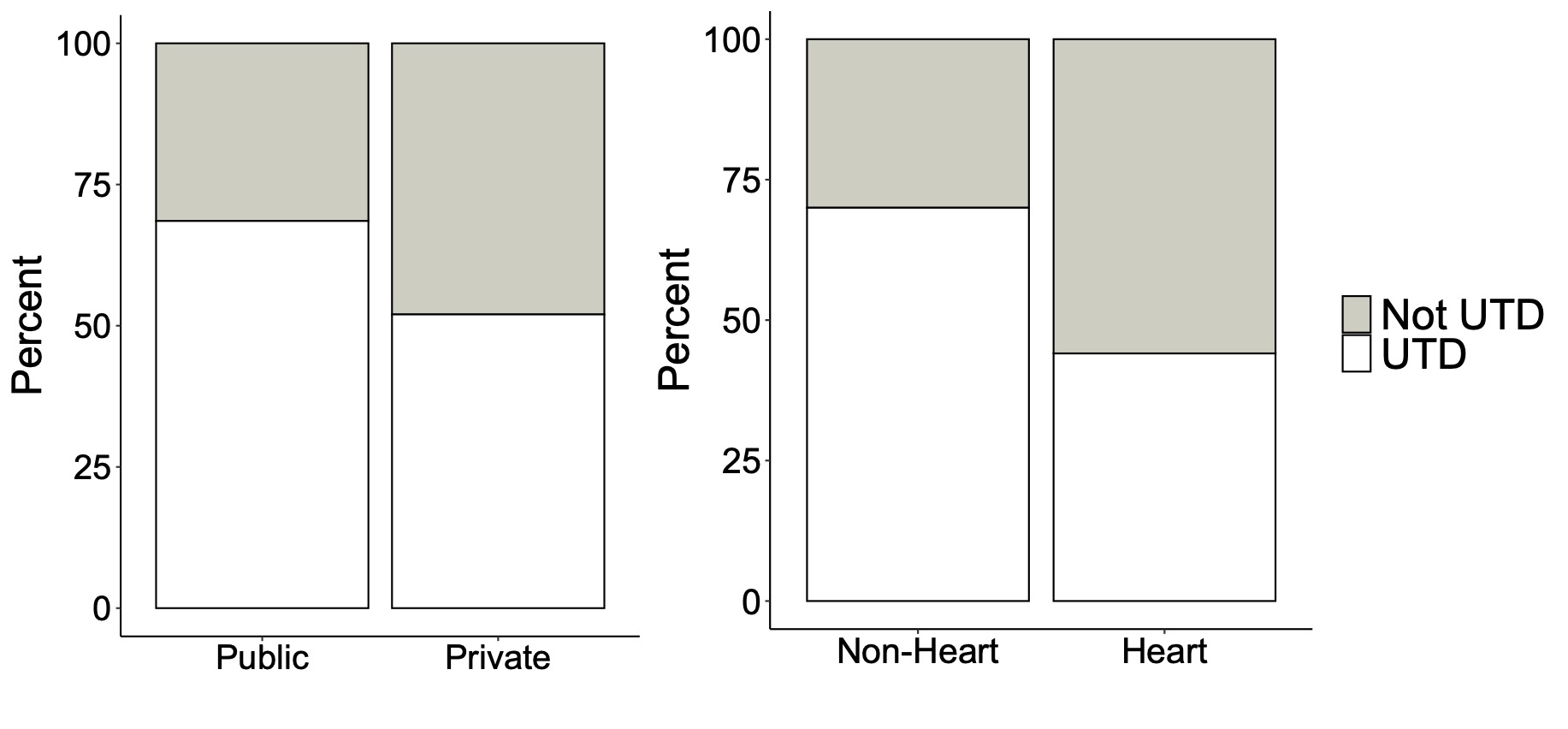

Supplement: Supplementary file 3 — Figure S3: Proportion of participants UTD for all childhood non‐live vaccines by type of transplant and insurance. The proportion of participants UTD vs. not UTD on all 6 routine non‐live childhood vaccines among heart vs. non‐heart transplant recipients (left panel) and participants with public vs. private insurance (right panel) is shown above. Participants were considered UTD if they met criteria for being UTD for each of the 6 non‐live vaccines. Participants were considered not UTD if they failed to meet criteria for being UTD for at least 1 of the 6 non‐live vaccines. [file PETR-30-e70302-s001.jpg]
