# Supplementary material for: Sociodemographic and Clinical Factors Impact Non‐Live Vaccine Coverage After Pediatric Solid Organ Transplantation: A Single Center Study
Source: Pediatr Transplant. 2026 Mar 23;30(3):e70302. doi: 10.1111/petr.70302 (PMC13009305; doi:10.1111/petr.70302)
Supplement: Supplementary file 4 — Table S1: Definitions of eligibility and completeness for non‐live vaccines. [file PETR-30-e70302-s004.docx]

| **Supplemental Table 1. Definitions of eligibility and completeness for non-live vaccines.** | | | |
| --- | --- | --- | --- |
| **Vaccine** | **Eligible** | **UTD**^†^ | |
| DTaP | 1. Participants transplanted prior to 1 year of age 2. Participants less than 4 years of age with less than four total doses of DTaP 3. Participants between 3 and 4 years of age who had received at least four doses of DTaP 4. Participants at least 4 years of age who had not received a dose of DTaP (or Tdap) after their 4^th^ birthday | | 1. Participants less than 1 year of age who received three doses of DTaP 2. Participants less than 4 years of age who received four doses of DTaP 3. Participants who received four or five doses of DTaP prior to turning 6 years of age, provided one dose was administered after their 4^th^ birthday 4. Participants greater than 7 years of age who received a dose of Tdap as a catch-up vaccine |
| Hep A | 1. Participants who had not received two doses of Hep A prior to transplant | | 1. Participants who had not received any dose of Hep A but were less than 1 year of age at the end of the study period 2. Participants greater than 1 year of age who received two doses of Hep A |
| Hep B | 1. Participants transplanted prior to being 6 months of age 2. Participants greater than 6 months of age who had not received three doses of Hep B | | 1. Participants greater than 6 months of age who received three doses of Hep B |
| Hib | 1. Participants transplanted prior to being 1 year of age 2. Participants between 12 and 15 months of age who had not received at least three total doses of Hib, including one dose administered after their 1^st^ birthday or had not received two total doses after their 1^st^ birthday 3. Participants between 15 months and 5 years of age who had not received any dose of Hib | | 1. Participants greater than 1 year of age who received at least three doses of Hib with at least one dose administered after their 1^st^ birthday OR who received at least two doses of Hib after their 1^st^ birthday 2. Participants who received one dose of Hib after turning 15 months of age 3. Participants greater than 5 years of age who had never received a dose of Hib |
| HPV | 1. Participants at least 10 years of age who had not received any doses of HPV prior to transplant 2. Participants who had received one dose of HPV prior to their 15^th^ birthday 3. Participants who had received less than three doses of HPV after their 15^th^ birthday | | 1. Participants less than 13 years of age who had not received any dose of HPV 2. Participants who received two doses of HPV prior to their 15^th^ birthday 3. Participants who received three doses of HPV after their 15^th^ birthday |
| IPV | 1. Participants transplanted prior to being 1 year of age 2. Participants less than 4 years of age with less than three total doses of IPV 3. Participants between 3 and 4 years of age who had received at least three doses of IPV 4. Participants at least 4 years of age who had not received a dose of IPV after their 4^th^ birthday | | 1. Participants less than 1 year of age who received two doses of IPV 2. Participants between 1 and 6 years of age who received at least three doses of IPV 3. Participants greater than 6 years of age who received at least three total doses of IPV, provided one dose was administered after their 4th birthday |
| MCV | 1. Participants at least 10 years of age who had not received any doses of MCV prior to transplant 2. Participants who were at least 15 years of age and had received one dose of MCV prior to transplant | | 1. Participants less than 13 years of age who had not received any dose of MCV 2. Participants less than 17 years of age who had received at least one dose of MCV 3. Participants who received their first dose of MCV after their 16^th^ birthday |
| PCV | 1. Participants transplanted prior to being 1 year of age 2. Participants between 1 and 2 years of age who had not received at least three total doses of PCV, including one dose administered after their 1^st^ birthday OR had not received two total doses after their 1^st^ birthday 3. Participants greater than 2 years of age who had not received any dose of PCV | | 1. Participants greater than 1 year of age who received at least three doses of PCV with at least one dose administered after their 1^st^ birthday OR who received at least two doses of PCV after their 1^st^ birthday 2. Participants who received one dose of PCV after their 2^nd^ birthday |
| ^†^UTD, up to date; For all vaccines, participants were considered UTD if a catch-up schedule was started but the study period ended prior to the minimum interval for the next dose | | | |
