# Supplementary material for: Sociodemographic and Clinical Factors Impact Non‐Live Vaccine Coverage After Pediatric Solid Organ Transplantation: A Single Center Study
Source: Pediatr Transplant. 2026 Mar 23;30(3):e70302. doi: 10.1111/petr.70302 (PMC13009305; doi:10.1111/petr.70302)
Supplement: Supplementary file 5 — Table S2: UTD vaccines by transplant type. [file PETR-30-e70302-s002.docx]

| **Supplemental Table 2. UTD vaccines by transplant type** | | | | |
| --- | --- | --- | --- | --- |
|  | **Heart**  **N=59** | **Intestine**  **N=10** | **Kidney**  **N=34** | **Liver**  **N=96** |
| DTaP | 47/59 (80%) | 10/10 (100%) | 32/34 (94%) | 84/96 (88%) |
| Hep A | 42/58 (72%) | 10/10 (100%) | 28/34 (82%) | 75/95 (79%) |
| Hep B | 42/59 (71%) | 9/10 (90%) | 28/34 (82%) | 83/96 (86%) |
| Hib | 57/59 (97%) | 10/10 (100%) | 34/34 (100%) | 88/96 (92%) |
| IPV | 50/59 (85%) | 10/10 (100%) | 32/34 (94%) | 87/96 (91%) |
| PCV | 55/59 (93%) | 10/10 (100%) | 32/34 (94%) | 87/96 (91%) |
| MCV^†^ | 25/33 (76%) | 3/4 (75%) | 14/24 (58%) | 15/26 (58%) |
| HPV^†^ | 18/31 (58%) | 2/3 (67%) | 15/23 (65%) | 15/26 (58%) |
| Each vaccine was assessed independently  ^†^Participants who were between 11-13 years of age but had not received any doses of the respective vaccine were excluded | | | | |
